# Effect of Temperature on Isolation and Characterization of Hydroxyapatite from Tuna *(Thunnus obesus)* Bone

**DOI:** 10.3390/ma3104761

**Published:** 2010-10-15

**Authors:** Jayachandran Venkatesan, Se Kwon Kim

**Affiliations:** Department of Chemistry, Pukyong National University, Busan 608-737, Korea; E-Mail: venkatjchem@gmail.com (J.V.); Marine Bioprocess Research Center, Pukyong National University, Busan 608-737, Korea

**Keywords:** *Thunnus obesus*, thermal calcinations, hydroxyapatite, X-ray diffraction, cytotoxicity

## Abstract

The effect of temperature on isolation and characterization of hydroxyapatite (HAp) from tuna bone was evaluated at different temperatures ranging from 200 °C to 1200 °C. The calcined bones were characterized by thermo gravimetric analysis (TGA), Fourier transform infrared spectroscopy (FTIR), X-ray diffraction (XRD), field emission scanning electron microscopy (FE-SEM), energy dispersive X-ray spectroscopy (EDX) and cytotoxicity assay. The FTIR and TGA results revealed the presence of inorganic and organic matrices in raw bone and a preserved carbonated group in the derived HAp. The XRD results of the derived HAp were coherent with the Joint Committee on Powder Diffraction Standards (JCPDS-09-0432/1996) data. In addition, FE-SEM results revealed the formation of nanostructured HAp (80–300 nm) at 600 °C and crystal agglomeration was observed with an increase in temperature. The calcium to phosphorous weight ratio was determined by EDX results of treated bones. Derived HAp with various crystal sizes had no cytotoxicity on the MG 63 cell line. Based on the analysis, we conclude that varying the isolation temperature between 600–900 °C has tremendous impact on the production of HAp from *Thunnus obesus* with required properties.

## 1. Introduction

Bone is a hierarchical structure and is made up of carbonated hydroxyapatite, type I collagen, non collagenous proteins and water. The average mineral content of bone tissue is species dependent and lies within the range of 50 to 74 wt % [1]. Hydroxyapatite (HAp) [Ca_10_(PO_4_)_6_(OH)_2_], is considered to play a vital role in various fields including the replacement of bone tissue [2], reconstruction of skull defects [3], tissue engineering [4], artificial bone synthesis [5,6,7], biosensor [8], removal of heavy metals [9], and as drug carrier [10]. HAp can be derived either from a natural source or by a synthetic method. Although good results have been obtained through several synthetic methods like hydrothermal [11,12], emulsion liquid membrane [13], precipitation [14,15], radio frequency thermal plasma [16], ultrasonic precipitation [17], reverse micro emulsion [18], sol-gel [19,20,21] and polymer assisted method [22]; these methods are rather complicated and necessitate a biologically hazardous process involving the evolution of ammonia. Moreover, chemical methods are time consuming processes (e.g., sol-gel synthesis), and gelation/aging, drying and sintering also require precisely controlled reaction conditions [22].

On the other hand, the production of HAp from natural sources is inexpensive and uncomplicated. The thermal calcination method is commonly used for the isolation of natural HAp. Tuna (*Thunnus obesus*) is a fish species of great commercial importance in the tropical and subtropical waters of the Pacific, Atlantic and Indian oceans [23]. Specifically, *Thunnus obesus* occupies 12% of the total amount of fish production in Korea (Production Database of Ministry of Maritime Affairs and Fisheries of Korea). It is usually processed as canned food and sliced raw meat in a factory and the by-products of tuna are affluent and collected at once [24]. The waste of *Thunnus obesus* has recently become a serious issue in coastal areas of Korea; one of the simplest ways to decrease pollution is the selective isolation of HAp from this waste. Ozawa *et al*., has reported the removal of aqueous chromium by fish bone waste originated HAp [25]. Micro structural HAp has already been obtained from fish bone by thermal treatment [26,27], thermal decomposition, alkaline hydrothermal, sub critical water process from bovine bone [28,29,30], teeth and bones of pig, extracted human teeth [31] and cuttle fish [32]. Although much has been learned about HAp isolation from natural sources [33], the most important parameter, exact isolation temperature, remains poorly understood.

In the present study, we have utilized *Thunnus obesus* bone as a new marine source for isolation of HAp and subjected the bone to various physiochemical properties, in order to find out the optimum conditions for HAp isolation. The derived HAp can be used for various medical and industrial applications and substantially increases the economical value of *Thunnus obesus* bone.

## 2. Results and Discussion

### 2.1. General Description

The removal of the organic portion was observed at different temperatures with changes in the color of the bone (Table 1). The color of the raw *Thunnus obesus* bone was observed as light yellow, which consequently changed into black, tan and off-white when subjected to calcination at 300 °C, 400 °C, 500 °C and 600 °C temperatures, respectively. The color of the *Thunnus obesus* bone turned white with further increase in the temperature. The different colors observed below 600 °C revealed the association of the organic matrix within the bone. It is evident from the results that different degrees of removal of organic portion were observed at varying temperatures, with 600 °C being the optimal temperature to produce HAp with almost no organic substances associated. The isolation yield of HAp at different temperature 600 °C, 900 °C and 1200 °C were 62.12%, 59.33% and 57.64%, respectively.

**Table 1:** Residues and color of calcined *Thunnus obesus* bone.

Sample no.	Calcination Temperature (°C)	Calcination Period in (h)	Initial Weight (g)	After calcination (g)	Residue (%)	Description
1	1200	5	2.0000	1.1527	57.6350	white
2	1100	5	2.0020	1.1529	57.5874	white
3	1000	5	2.0024	1.1771	58.7845	white
4	900	5	2.0011	1.1872	59.3274	white
5	800	5	2.0030	1.1936	59.5906	white
6	700	5	2.0032	1.2129	60.5481	off white
7	600	5	2.0017	1.2434	62.1172	off white
8	500	5	2.0052	1.2688	63.2755	Tanish
9	400	5	2.0031	1.3402	66.9063	Tanish
10	300	5	2.0061	1.5162	75.5795	Black
11	200	5	2.0000	1.7360	86.8000	Black
12	Raw fish bone	-	-	-	-	Yellow

### 2.2. Thermal Analysis of *Thunnus obesus* Bone

**Figure 1:**
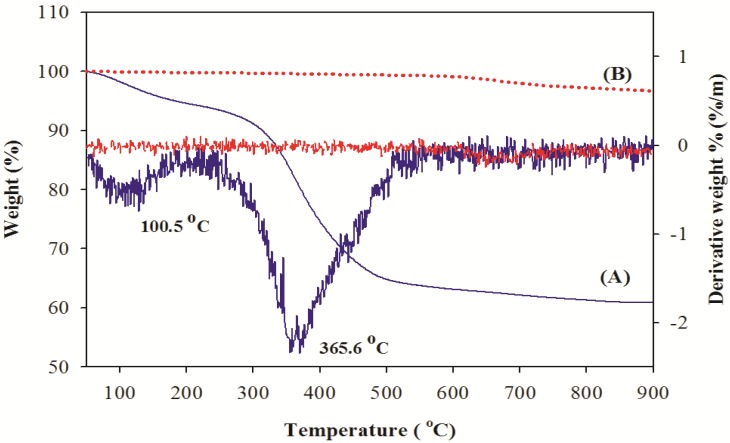
TGA curves of (**A**) raw fish bone and (**B**) thermal treatment at 900 °C.

The removal of the organic portion from *Thunnus obesus* bone and derived HAp (900 °C) was confirmed by TGA and DTG analysis and results are shown in Figure 1(A), (B). In the TGA and DTG curves, two inflection points were observed in the *Thunnus obesus* bone at 100.5 °C and 365.6 °C which corresponds to removal of the water and organic matter. No significant weight loss was observed between 600 °C and 900 °C, indicating the complete removal of organic materials such as collagen, lipids, chondroitin sulfate, and keratin sulfate below 600 °C. The average amount of water and organic phase removed during heat treatment was calculated with DTG analysis, and it was found that approximately 4.76% water and 30.02% organic matter was removed. Therefore, it can be inferred that about 35% of total weight loss was due to removal of water and organic substances from the *Thunnus obesus* bone when it was subjected to heat treatment up to 600 °C. Figure 1(B) depicts the derived HAp at 900 °C, no inflection points were observed from the starting temperature. This significantly indicated that HAp derived at 900 °C lacks organic moieties and water which further confirms that it is in the pure form.

### 2.3. FT-Infrared Spectroscopic Analysis

**Figure 2:**
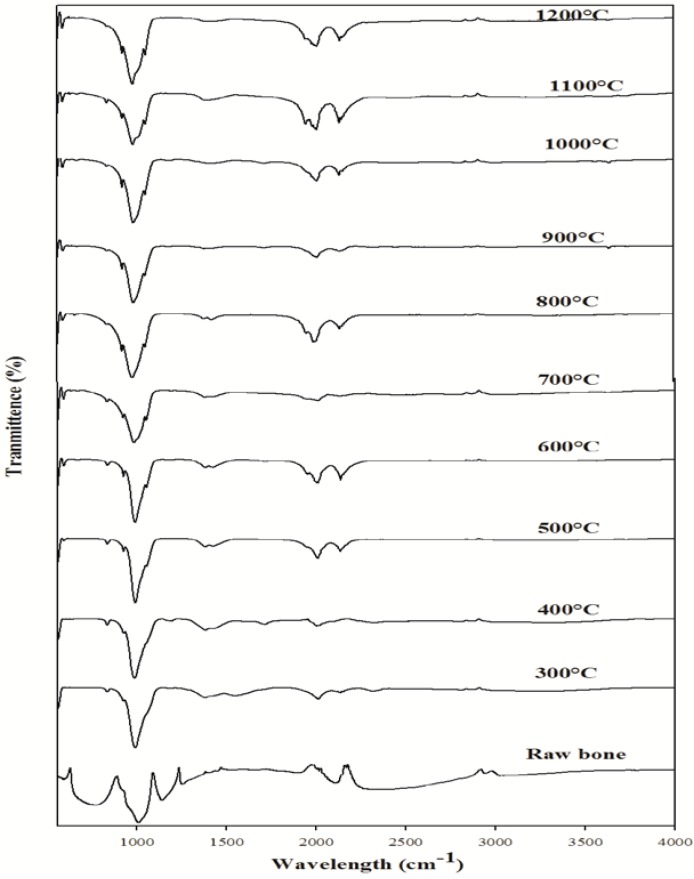
FTIR spectra of *Thunnus obesus* bone calcined from 300 °C to 1200 °C.

FT-IR is a promising tool to identify unknown substances and to determine the amount of components in a given sample. This test was performed to get authenticated information about the vibrational origin of the phosphate, carbonate and amide groups and to confirm the production of HAp with no association of organic moieties. The FT-IR spectrum of raw *Thunnus obesus* bone and derived HAp at different temperatures are shown in Figure 2. The FT-IR spectrum of *Thunnus obesus* bone calcined between temperatures of 500 °C to 1200 °C revealed the only characteristic peak of HAp, which is consistent with some previous reports [29,34,35]. A large number of bands in the spectra (601, 631, 873, 962, 962, 1027, 1088, 1413, 1454, 2034, 2157 cm^−1^ and a broad band observed between 3300–3600 cm^−1^) matched the bands in the HAp reference spectrum and are in close agreement with the same [29,30]. At lower temperatures (300 °C and 400 °C), the peak corresponding to the phosphate (PO_4_^3−^) group at 1088 cm^−1^ was not observed and it appeared only at temperatures above 500 °C. This may be due to the removal of all the organic material from the raw *Thunnus obesus* bone and formation of HAp crystals.

Thermal stability is an important feature of derived HAp. Ooi *et al*., have reported the formation of calcium phosphate when the bovine bone is subjected to higher temperatures, which shows that the derived HAp is not thermally stable [30]. On the contrary, no phase transformation due to calcination was observed in our study even at high temperatures (500 °C to 1200 °C). Subsequently, peaks at 1026–1088 cm^−1^ and 962 cm^−1^ are attributed to PO_4_^3−^. Additionally; all the FT-IR spectra samples exhibited a peak at 632 cm^−1^ and a broad peak at 3300–3600 cm^−1^ due to the presence of the hydroxyl group. The intense peaks observed at 1413 and 1457 cm^−1^ in the spectrum of calcined *Thunnus obesus* bone are attributed to CO_3_^2−^.

### 2.4. X-Ray Diffraction Analysis of *Thunnus obesus* Bone

**Figure 3:**
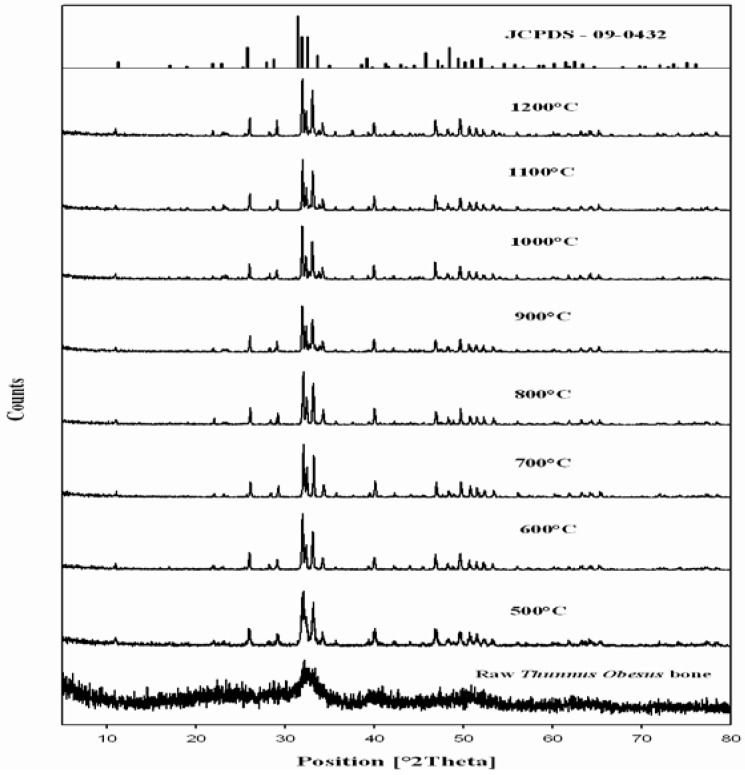
XRD results of *Thunnus obesus* bone from 500 °C to 1200 °C.

The phase and purity of derived HAp crystals were confirmed with XRD analysis. Figure 3 shows the XRD pattern of raw *Thunnus obesus* bone and treated bone at different temperatures. The present XRD results suggest that the HAp stability in the bone matrix was not disrupted when calcined in air up to 1200 °C, as the chemical structure of HAp has not been affected and no other peak was obtained apart from HAp. The crystalline composition of calcined *Thunnus obesus* bone was found to be similar to that of HAp (JCPDS-09-0432/1996) when calcined between 700–1000 °C. It is well known that as the temperature increases the intensity of the peak increases with a decrease in the peak width [35]. The intensity of the raw *Thunnus obesus* bone was found to be dispersed by x-ray radiation with a lowered intensity and wider peak. This may be due to the presence of extracellular matrix and fibrous proteins. When subjected to calcination at higher temperatures, the subsequent peaks were highly intense and sharp, indicating the removal of organic portion [36]. Table 2 shows the d-spacing line (estimated by Bragg law), 2θ angle and relative intensity at the strongest peak in the XRD spectra. The obtained d-spacing lines, 2θ angle, relative intensity at the different temperatures, have been compared with standard HAp (JCPDS-09-0432/1996) and the error was estimated at every plane. From these results, it is evident that HAp derived at different temperatures is very close to the standard HAp in purity and stability.

**Table 2:** d-planar spacing, 2θ angle and relative intensity of obtained HAp using thermal decomposition method; the results are compared with the standard HAp (JCPDS-09-0432).

h k l	d-spacing (nm)					Position (2θ)					Intensity (%)				
	JCPDS	700 °C	800 °C	900 °C	1000 °C	JCPDS	700 °C	800 °C	900 °C	1000 °C	JCPDS	700 ° C	800 °C	900 °C	1000°C
0 0 2	0.344	0.341	0.342	0.343	0.342	25.87	26.11	26.08	26.01	25.98	40	29.9	34.6	29.2	27.5
2 1 1	0.281	0.279	0.280	0.280	0.280	31.77	32.05	32.00	31.91	31.89	100	100	100	100	100
1 1 2	0.278	0.275	0.276	0.277	0.277	32.19	32.45	32.41	32.34	32.32	60	49.1	56.9	48.3	40.1
3 0 0	0.272	0.269	0.270	0.271	0.271	32.90	33.19	33.14	33.06	33.04	60	85.2	84.4	77.4	71.2
2 0 2	0.263	0.261	0.262	0.262	0.262	34.04	34.32	34.27	34.23	34.19	25	24.6	28	23.5	19.6
3 1 0	0.226	0.224	0.225	0.226	0.225	39.81	39.47	39.39	39.96	39.93	20	27.9	26.8	30	25.5
2 1 3	0.184	0.183	0.184	0.184	0.183	49.46	49.71	49.69	49.63	49.85	40	26.3	29.3	28.2	23.9
**Error**		**0.047**	**0.038**	**0.024**	**0.019**		**0.056**	**0.051**	**0.031**	**0.031**		**1.6**	**1.3**	**1.7**	**1.7**

The relative intensity of calcined bone was found to be closest to standard HAp at 800 °C. The 2θ angles varied a little in comparison to standard HAp, which might be due to the trace removal of OH radicals. According to Wang and Chaki [37], dehydroxylation of the HAp phase would cause a small degree of peak shifting in the XRD trace. In the present work, it was found that XRD 2θ positions of the bone samples calcined at 700 °C and 1000 °C shifted by total error of 0.056 and 0.031, respectively, thus indicating that the HAp lattice has contracted due to loss of OH radicals. It should be noted that although the decomposition of HAp phases was not detected in samples calcined at 1000 °C, this can be observed by simply comparing the XRD peak’s position which correspond to the higher intensities planes, (0 0 2), (2 1 1), (1 1 2), (3 0 0), (2 0 2) (3 1 0) and (2 1 3) of calcined *Thunnus obesus* bone.

### 2.5. Field Emission-Scanning Electron Microscope Analysis

**Figure 4:**
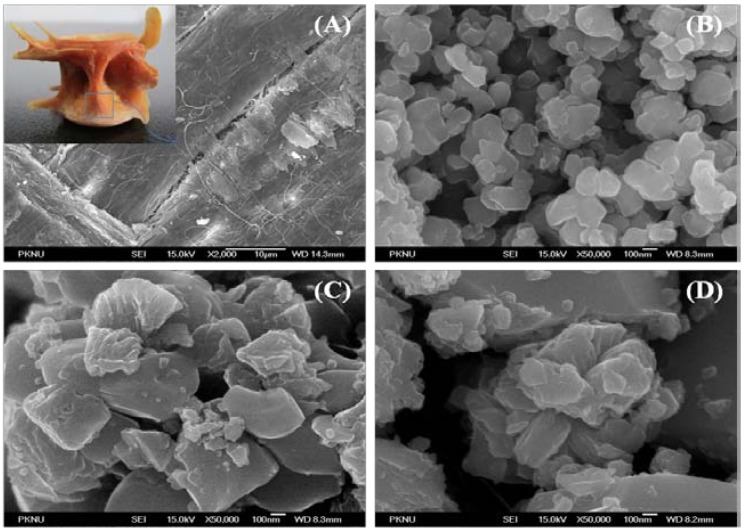
SEM results of (**A**) raw fish bone and treated at (**B**) 600 °C, (**C**) 900 °C, (**D**) 1200 °C. Inset shows the whole picture of raw vertebral *Thunnus obesus* bone.

The surface morphology and crystal size of the derived HAp were studied under FE-SEM. Figures 4 (A), (B), (C) and (D) show FE-SEM pictures of raw bone derived HAp at 600 °C, 900 °C and 1200 °C, respectively. Microcrystal of HAp in the natural bone is very small, with a crystalline size of 5–10 nm, 10–15 nm wide and more than a few micrometers long [26,36]. The microstructures of raw *Thunnus obesus* bone appeared to be dense due to the presence of organic substances shown in Figure 4(a). In Figure 4(B), formation of nanoparticles was clearly evident in the derived HAp at 600 °C with crystal sizes 80–300 nm. Whereas, in Figure 4 (C) and (D), HAp microstructures were observed with increase in temperature from 900 °C to 1200 °C. The crystal size of derived HAp at higher temperatures (900 °C and 1200 °C) is 0.3–1.0 µm and 0.5–2.0 µm, respectively. It was conjectured from the surface morphology that the crystal size increases with respect to the temperature. The formation of these microstructures of derived HAp in the thermal process can be attributed to the tendency of particles to crystallize and agglomerate at high temperatures [28,38].

### 2.6. Electron Dispersive X-ray Analysis

**Figure 5:**
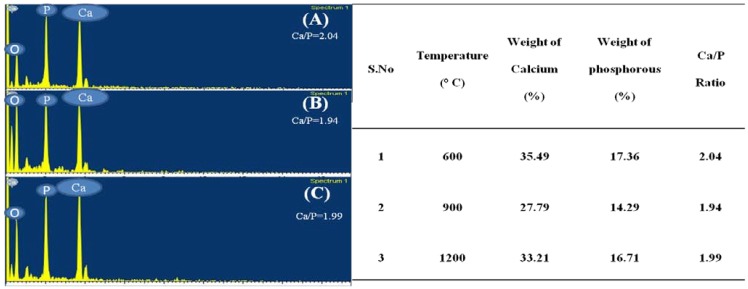
EDX results at (**A**) 600 °C, (**B**) 900 °C and (**C**) 1200 °C.

EDX is an analytical technique used for elemental analysis or chemical composition of a sample. Figure 5 (A), (B) and (C) represent the EDX data for derived HAp at 600 °C, 900 °C and 1200 °C, respectively. Based on the EDX signatures, the Ca/P weight ratio for derived HAp was calculated and was found to be 2.04, 1.94 and 1.99 at 600 °C, 900 °C and 1200 °C, respectively; the resultant values are consistent with previous values reported elsewhere. As the Ca/P weight ratio of the derived HAp at different temperatures did not show any considerable difference, it can be inferred that Ca/P weight ratio is independent of calcination temperature.

### 2.7. Cytotoxicity Assay

**Figure 6:**
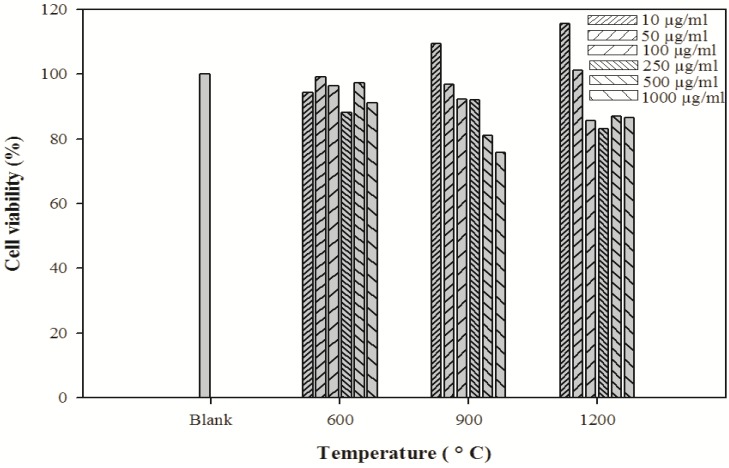
Cytotoxicity of HAp crystals at various temperatures on MG-63 cells. Culture cells were incubated under 5% CO_2_ and 37 °C and viable cells were detected by MTT assay.

The crystal size of HAp plays a major role in bone tissue engineering for nutrient supplementation and cell attachment. A highly porous and nanocrystal structure is a prerequisite to ensure that the biological environment is conductive for cell attachment, proliferation, tissue growth and adequate nutrient flow. The cytotoxicity effects of derived HAp crystals at different temperatures were investigated by MTT assay (Figure 6). The HAp crystals showed no cytotoxicity in the MG-63 cell line.

## 3. Experimental Section

### 3.1. Sample Preparation

The *Thunnus obesus* bones were washed carefully with hot water for two days in order to remove entire meat from the skin. The washed bones were mixed with 1.0% Sodium Hydroxide (NaOH) solution and acetone to remove proteins, lipids, oils and other organic impurities (the bone and NaOH solid/liquid ratio was maintained as 1:50). Then the bones were dried at 160 °C for 48 h and ground into 200 µm particle size. Further, 2 g of treated *Thunnus obesus* bone was placed in an open silica crucible and heated in an electric furnace (Dongwan Scientific Co. A/S. 051) 245–7521) under ambient conditions, at different temperatures ranging from 200 °C to 1200 °C with 5 h holding time.

### 3.2. Characterization

Thermal gravimetric analysis was achieved by the use of Pyris 7 TGA analyzer, Perkin Elmer Inc., U.S., with scan range from 50 to 900 °C, at constant heating rate of 10 °C min^−1^, with continuous nitrogen flow. The stretching frequencies (vibrational origin) of samples were examined by Fourier Transform Infrared Spectroscopy, Perkin Elmer (U.S.) and spectrum GX spectrometer within the range of 450 to 4000 cm^−1^. The phase and crystallinity of HAp were evaluated using X-ray diffractometer (PHILIPS X’Pert-MPD diffractometer, Netherlands) and Cu-Kα radiation of 1.5405 Å over a range of 5 to 80° angle, step size 0.02, scan speed 4°/min with 40 kV current and 30 mA voltage. The XRD resultant spectra were compared with literature profile JCPDS 09-0342/1996 to identify the compound. Morphology and chemical composition of HAp crystals was obtained by field emission scanning electron microscopy (FE-SEM JSM-6700F, JEOL, Japan) equipped with an *in situ* energy dispersive X-ray (EDX) spectrometer. MTT assay method was used to find out the cytotoxicity of derived HAp. For this, MG-63 cell line (Human osteosarcoma cell line) were cultured in DMEM medium supplemented with 5% fetal bovine serum, 2 mM glutamine and 100 µg/mL penicillin‑streptomycin and incubated at 37 °C in a humidified atmosphere with 5% CO_2_. The cells were grown at a concentration of 1 × 10^6^ cells/well in a 24 well plate. After 24 h, cells were washed with fresh medium and treated with various concentrations (10, 50, 100, 250, 500 and 1000 µg mL^−1^) of HAp crystals. After 24 h incubation, cells were rewashed with PBS; 2mL of MTT (1 mg mL^−1^) was added and further incubated for 4 h. Finally 2 mL of DMSO was added to solubilize the formazan salt formed and the amount of formazan salt was determined by measuring the OD at 570 nm using aGENios® microplate reader (Tecan Austria GmbH, Austria).

## 4. Conclusion

The present study shows the isolation of pure HAp from *Thunnus obesus* bone. Our work specifically signifies the potential use of thermally treated fish bone waste for the preparation of ceramics like HAp, which has a great potential as a viable and economical graft material in various medical and industrial applications. We conclude that calcination of raw bone at 600 °C to 900 °C led to the formation of pure HAp. The characterization results revealed that organic matter was fully removed from *Thunnus obesus* bone over 600 °C; rendering the derived HAp to be highly pure and suitable for further applications. Additionally, the crystalline nature of HAp was found to be directly proportional to the calcining temperature; the higher the temperature, the greater the particle size. When compared with the standard JCPDS-09-0432/1996 data, the total estimated error according to d-planar spacing and 2θ angle of derived HAp was less than 0.05% which confirms the purity of derived HAp by the thermal calcinations method. Moreover, the total error gradually decreased with increasing temperature, indicating that the purity of derived HAp gradually increases with increase in temperature. In addition to this, the derived HAp showed nanostructure (80–300 nm) crystals at low temperature (600 °C). The formation of microcrystals was observed at temperatures above 900 °C. It is easy to conclude here that temperatures above 900 °C may not be optimum for isolation of nanostructure HAp. The HAp crystals obtained at different temperatures were found to be nontoxic, irrespective of the crystal size, suggesting their safe utility in bone tissue engineering.

Based on the results of this study, we draw a conclusion that a temperature between 600–900 °C is optimum for isolation of HAp from *Thunnus obesus* with almost no organic portion, high purity, stability, crystallinity, nanostructure and no cytotoxicity; making it appropriate for use in biomedical applications.

## References

[1] Fratzl P., Gupta H., Paschalis E., Roschger P. (2004). Structure and mechanical quality of the collagen–mineral nano-composite in bone. J. Mater. Chem..

[2] Tang P., Li G., Wang J., Zheng Q., Wang Y. (2009). Development, characterization, and validation of porous carbonated hydroxyapatite bone cement. J. Biomed. Mater. Res. B.

[3] Staffa G., Nataloni A., Compagnone C., Servadei F. (2007). Custom made cranioplasty prostheses in porous hydroxyapatite using 3D design techniques: 7 years experience in 25 patients. Acta Neurochir..

[4] Nair M., Suresh Babu S., Varma H., John A. (2008). A triphasic ceramic-coated porous hydroxyapatite for tissue engineering application. Acta Biomater..

[5] Hirata A., Maruyama Y., Onishi K., Hayashi A., Saze M., Okada E. (2008). A Vascularized artificial bone graft using the periosteal fflap and porous hydroxyapatite; basic research and preliminary clinical application. Wound Repair Regen..

[6] Venkatesan J., Kim S.-K. (2010). Chitosan composites for bone tissue engineering—an overview. Mar. Drugs.

[7] Venkatesan J., Qian Z.J., Ryu B., Ashok Kumar N., Kim S.K. (2010). Preparation and characterization of carbon nanotube-grafted-chitosan—Natural hydroxyapatite composite for bone tissue engineering. Carbohyd. Polym..

[8] Salman S., Soundararajan S., Safina G., Satoh I., Danielsson B. (2008). Hydroxyapatite as a novel reversible in situ adsorption matrix for enzyme thermistor-based FIA. Talanta.

[9] Reichert J., Binner J. (1996). An evaluation of hydroxyapatite-based filters for removal of heavy metal ions from aqueous solutions. J. Mater. Sci..

[10] Kano S., Yamazaki A., Otsuka R., Ohgaki M., Akao M., Aoki H. (1994). Application of hydroxyapatite-sol as drug carrier. Bio-Med. Mater. Eng..

[11] Zhang H., Zhou K., Li Z., Huang S. (2009). Plate-like hydroxyapatite nanoparticles synthesized by the hydrothermal method. J. Phys. Chem. Solids.

[12] Chen J., Wang Y., Wei K., Zhang S., Shi X. (2007). Self-organization of hydroxyapatite nanorods through oriented attachment. Biomaterials.

[13] Jarudilokkul S., Tanthapanichakoon W., Boonamnuayvittaya V. (2007). Synthesis of hydroxyapatite nanoparticles using an emulsion liquid membrane system. Colloid. Surface. A.

[14] Sarig S., Kahana F. (2002). Rapid formation of nanocrystalline apatite. J. Cryst. Growth.

[15] Monmaturapoj N. (2008). Nano-size Hydroxyapatite Powders Preparation by Wet-Chemical Precipitation Route. J. Met. Mater. Miner..

[16] Xu J., Khor K., Dong Z., Gu Y., Kumar R., Cheang P. (2004). Preparation and characterization of nano-sized hydroxyapatite powders produced in a radio frequency (rf) thermal plasma. Mater. Sci. Eng. A.

[17] Cao L., Zhang C., Huang J. (2005). Synthesis of hydroxyapatite nanoparticles in ultrasonic precipitation. Ceram. Int..

[18] Guo G., Sun Y., Wang Z., Guo H. (2005). Preparation of hydroxyapatite nanoparticles by reverse microemulsion. Ceram. Int..

[19] Simon V., Lazar D., Turcu R., Mocuta H., Magyari K., Prinz M., Neumann M., Simon S. (2009). Atomic environment in sol–gel derived nanocrystalline hydroxyapatite. Mater. Sci. Eng. B.

[20] Liu D., Yang Q., Troczynski T., Tseng W. (2002). Structural evolution of sol–gel-derived hydroxyapatite. Biomaterials.

[21] Feng W., Mu-Sen L., Yu-Peng L., Yong-Xin Q. (2005). A simple sol–gel technique for preparing hydroxyapatite nanopowders. Mater. Lett..

[22] Tseng Y., Kuo C., Li Y., Huang C. (2009). Polymer-assisted synthesis of hydroxyapatite nanoparticle. Mater. Sci. Eng. C.

[23] Farley J., Clear N., Leroy B., Davis T., Mcpherson G. (2006). Age, growth and preliminary estimates of maturity of bigeye tuna, Thunnus obesus, in the Australian region. Mar. Freshwater Res..

[24] Cho S., Gu Y., Kim S. (2005). Extracting optimization and physical properties of yellowfin tuna (Thunnus albacares) skin gelatin compared to mammalian gelatins. Food Hydrocolloid..

[25] Ozawa M., Satake K., Suzuki R. (2003). Removal of aqueous chromium by fish bone waste originated hydroxyapatite. J. Mater. Sci. Lett..

[26] Ozawa M., Suzuki S. (2002). Microstructural development of natural hydroxyapatite originated from fish-bone waste through heat treatment. J. Am. Ceram. Soc..

[27] Haberko K., Bu ko M., Brzezi ska-Miecznik J., Haberko M., Mozgawa W., Panz T., Pyda A., Zar bski J. (2006). Natural hydroxyapatite—its behaviour during heat treatment. J. Eur. Ceram. Soc..

[28] Barakat N., Khil M., Omran A., Sheikh F., Kim H. (2009). Extraction of pure natural hydroxyapatite from the bovine bones bio waste by three different methods. J. Mater. Process. Tech..

[29] Joschek S., Nies B., Krotz R., Göpferich A. (2000). Chemical and physicochemical characterization of porous hydroxyapatite ceramics made of natural bone. Biomaterials.

[30] Ooi C. Y., Hamdi M., Ramesh S. (2007). Properties of hydroxyapatite produced by annealing of bovine bone. Ceram. Int..

[31] Dachun L., Wei C. (2007). Preparation and characterization of natural hydroxyapatite from animal hard tissues. Key Eng. Mat..

[32] Ivankovic H., Gallego Ferrer G., Tkalcec E., Orlic S., Ivankovic M. (2009). Preparation of highly porous hydroxyapatite from cuttlefish bone. J. Mater. Sci.-Mater. Med..

[33] Kim S., Park P., Kim Y. (2001). Study on acute subcutaneous toxicity of hydroxyapatite sinter produced from tuna bone in Sprague—Dawly rats. Korean J. Life Sci..

[34] Walters M., Leung Y., Blumenthal N., LeGeros R., Konsker K. (1990). A Raman and infrared spectroscopic investigation of biological hydroxyapatite. J. Inorg. Biochem..

[35] Koutsopoulos S. (2002). Synthesis and characterization of hydroxyapatite crystals: A review study on the analytical methods. J. Biomed. Mater. Res..

[36] Glimcher M. J. (1959). Molecular biology of mineralized tissues with particular reference to bone. Rev. Mod. Phys..

[37] Wang P., Chaki T. (1993). Sintering behaviour and mechanical properties of hydroxyapatite and dicalcium phosphate. J. Mater. Sci.-Mater. Med..

[38] Han Y., Li S., Wang X., Jia L., He J. (2007). Preparation of hydroxyapatite rod-like crystals by protein precursor method. Mater. Res. Bull..

